# Chemical Composition, Antimicrobial and Antioxidant Activities of Essential Oils from Two *Avicennia schaueriana* Stapf & Leechm. Ex Moldenke (Acanthaceae) Populations

**DOI:** 10.3390/medicines4020026

**Published:** 2017-05-01

**Authors:** Kamilla N. Machado, Telma M. Kaneko, Maria Cláudia M. Young, Cynthia Murakami, Inês Cordeiro, Paulo Roberto H. Moreno

**Affiliations:** 1 Programa de Pós-Graduação em Fármaco e Medicamentos-Faculdade de Ciências Farmacêuticas, Universidade de São Paulo, São Paulo 05508-000, Brasil; kamillan@iq.usp.br (K.N.M.); tsakuda@usp.br (T.M.K.); 2 Instituto de Química, Universidade de São Paulo, São Paulo 05508-000, Brasil; 3 Instituto de Botânica do Estado de São Paulo, São Paulo 04301-902, Brasil; marxyoungmc@gmail.com (M.C.M.Y.); cynmurakami@gmail.com (C.M.); isandona@uol.com.br (I.C.)

**Keywords:** *Avicennia*, mangrove plant, essential oil, chemotypes, antioxidant activity, antimicrobial activity, GC-MS

## Abstract

**Background:** *Avicennia schaueriana* Stapf & Leechm. ex Moldenke (Acanthaceae) is a native species from the Brazilian mangroves presenting ecological and economic significance. This study compared the composition and the biological activities from the essential oils obtained from two *A. schaueriana* populations collected at Jureia-Itatins and Ilha do Cardoso. **Methods:** Essential oils were obtained by conventional means, and their compositions were analyzed by GC-MS. Screening assays for antimicrobial activity were carried out by the microdilution method and the antioxidant potential was assessed by the DPPH scavenging method. **Results:** The GC-MS analysis indicated that the Jureia oil (**1**) was composed mostly of the fatty acids palmitic (46.5%) and myristic (11.6%) acids, while the main components for the Ilha do Cardoso oil (**2**) were eugenol (19.7%), eugenol acetate (12.9%) and palmitic acid (15.1%). The oils showed an IC_50_ of 0.9 ± 0.011 mg/mL for **1** and 1.13 ± 0.028 mg/mL for **2** in the DPPH assay. The antimicrobial assay indicated MIC > 217 µg/mL for all tested microorganisms. **Conclusions:** The different essential oil composition may indicate the presence of chemotypes for *A. schaueriana*. The antioxidant activity of the oils was weak if compared with flavonoids. Despite the high MIC values, these oils presented some antibacterial potential against *Pseudomonas aeruginosa*.

## 1. Introduction

*Avicennia* L. (Acanthaceae) comprises eight species of mangrove trees that occur in intertidal zones of estuaries and seashores. These species are found in tropical and subtropical coastlines, and they have ecological and economic significance [1]. The genus includes true mangrove plants that have been reported to contain iridoids glycosides, flavonoids, diterpenes, triterpenes, fatty acids, and naphthoquinones [1,2,3,4,5,6,7]. *Avicennia* spp. are used in folk medicine to treat various diseases such as scabies, hepatitis, leprosy, burns, snake bites, tumors, ulcers, rheumatism, sore throat, pustule, and others skin diseases [8]. A naphthoquinone isolated from *A. germinas* L. and a flavonoid isolated from *A. marina* (Forsk.) Vierh. demonstrated cytotoxic activity against human cancer cell lines [9,10]. Additionally, *A. marina* extracts showed antimicrobial activity against pathogenic bacterial and fungal strains [11].

*A. schaueriana* Stapf & Leechm ex. Moldenke, also called black mangrove or siriuba, is one of the native Brazilian mangrove species. The antimicrobial activity of *A. schaueriana* extracts has been reported [2,8]. However, essential oils from this species have never been evaluated for its chemical composition or biological activities. In the genus, only the *A. marina* leaf and fruit oils have been analyzed, presenting 2,6-bis(1,1-dimethylethyl)-4-methylphenol (BHT) (41.91%) and 2-phenyl-1,3-butadiene (24.56%) as major components for the leaf oils, and methyl palmitate (41.9%), methyl *p*-vinylbenzoate (9.68%) and methyl ester (*9Z*,15*Z*)-9,15-octadecadienoic acid (8.28%) for the fruit oils [12,13]. Hence, this study aims to evaluate the composition of leaf essential oils from two *A. schaueriana* specimens and to compare their in vitro antimicrobial and antioxidant activities.

## 2. Materials and Methods 

### 2.1. Plant Material 

*Avicennia schaueriana* Stapf & Leechm (Acanthaceae) leaves were collected at Parque Estadual da Ilha do Cardoso, São Paulo, Brazil (23°03’ to 25°19’ S and 47°53’ to 48°30’ W) and at Estação Ecológica Jureia-Itatins, São Paulo, Brazil (24°17’ to 24°35’ S and 47°00’ to 47°31’ W), from five individual plants in each location. The botanical identity was confirmed by Dr. Inês Cordeiro (Instituto de Botânica, São Paulo, Brazil). The voucher specimens were deposited in the Herbarium of the Instituto de Botânica, São Paulo, Brazil, with vouchers RSCabral 16-SP for the plant collected at Parque Estadual da Ilha do Cardoso and RSCabral 46 for the one collected at Estação Ecológica Jureia-Itatins. 

### 2.2. Extraction of the Essential Oil

*A. schaueriana* leaves collected at Parque Estadual da Ilha do Cardoso (218.92 g) and Estação Ecológica Jureia-Itatins (241.12 g) were pooled and dried in a circulating air oven at 40 °C for 24 h. The essential oil was obtained from each population pool by hydrodistillation for 4 h in a *Clevenger*-type apparatus. The essential oil was dried over anhydrous sodium sulfate and stored in a glass flask at −22 °C until analysis by GC-MS. The yields were calculated based on the dry weight of each plant. 

### 2.3. GC-MS Analysis 

Qualitative analysis of essential oils from the leaves of *A. schacueriana* collected at Parque Estadual da Ilha do Cardoso and Estação Ecológica da Jureia-Itatins were performed in an Agilent6890 Series GC (Agilent, Santa Clara, CA, USA) interfaced with a 5973 series quadrupole MS detector (Agilent, Santa Clara, CA, USA), equipped with a DB-5 column (30 m × 0.25 mm i.d. × 0.25 μm) (Agilent J&W, Santa Clara, CA, USA). Chromatography conditions were as follows; over temperature: initially held at 40 °C for 1 min and subsequently increased to 240 °C at 3 °C/min; carrier gas: He at a flow rate of 1 mL/min; injector and detector temperature: 250 °C, electron ionization: 70 eV. The components were identified by comparing retention indices (evaluated in relation to the retention times of a series of *n*-alkanes) and mass spectra with those reported in the literature [14,15]. 

### 2.4. In vitro Antioxidant Activity 

The antioxidant activity of the essential oils was evaluated by the 2.2-diphenyl-2-picrylhydrazyl hydrate (DPPH) radical scavenging method. The essential oil solubilized in methanol at concentrations between 435–13.60 µg/mL in a microplate well (sample), pure methanol (blank), and DPPH solution (202.88 µmol, control) were transferred to a 96-well microplate (Corning, New York, NY, USA). The sample solubilized in methanol without DPPH was used as a blank sample. The plate was incubated in the dark for 30 min at room temperature. Then, the absorbance was read at the wavelength of 520 nm, using a multi-well scanning spectrophotometer (Synergy HT Biotek, Winooski, VT, USA). The radical scavenging capacity (expressed as percentage) was calculated as the rate between [(Abs control − Abs blank) − (Abs sample − Abs blank sample)/(Abs control − Abs blank)] × 100. Quercetin was employed as a positive control. The amount of extract required to reduce the initial DPPH concentration in the reaction by 50% is referred to as the inhibitory concentration (IC_50_). The data were compared by one-way Anova, followed by Tukey’s test, representing the mean and confidence interval (CI_95_) for *n* = 3. The results were considered statistically different when *p* < 0.05. 

### 2.5. Antimicrobial Activity

Antifungal activity was assessed against *Aspergillus brasiliensis* (ATCC 16404) and *Candida albicans* (ATCC 10231), and antibacterial activity was evaluated against one model of Gram-positive bacteria, *Staphylococcus aureus* (ATCC 6538), and two models of Gram-negative bacteria, *Pseudomonas aeruginosa* (ATCC 9027) and *Escherichia coli* (ATCC 8739). Microdilution in broth was the method used to determine the antimicrobial activity. The growth inhibitions were determined by broth microdilution method according to the protocols of the Clinical and Laboratory Standards Institute [16,17]. Microorganisms were incubated for 48 h at 28 °C on SDA (for the yeast and fungi) or for 24 h at 37 °C on TSA (for the bacteria). Following incubation, a suspension of the microorganism was prepared and standardized in saline solution (0.9%) for subsequent inoculation into liquid culture medium (SDB for the yeast and fungi; TSB for the bacteria) to give a final concentration of 2 × 10^3^ colony-forming units (CFU)/mL in each well of the microplate. Samples of the essential oil of each plant collected were diluted in dimethyl sulfoxide:methanol 1:1 (*v*/*v*) to give a final concentration of 217 μg/mL in each microplate well. After incubation, the growth inhibition was measured by reading the absorbance at a wavelength of 630 nm, using a multi-well scanning spectrophotometer (Synergy HT Biotek, Winooski, VT, USA); except for *A. brasiliensis*, where the growth was evaluated only visually. Growth inhibitions (%) were determined for samples that inhibited the growth of the microorganism in the microplate well test.

## 3. Results

### 3.1. Chemical Composition of Essential Oil

The essential oil yields (*w*/*w*) for the *A. schaueriana* leaves were 0.0035% for the specimen collected at the Estação Ecológica Jureia-Itatins (**1**) and 0.0085% for that collected at Parque Estadual da Ilha do Cardoso (**2**). The GC-MS analysis indicated that the oils were composed of at least 27 compounds, of which 25 were identified. The compounds, their retention indices, and the percentage of each constituent for both essential oils are listed in Table 1. The full mass spectra from the unidentified compounds are provided as supplementary material (Figures S1–S10).

### 3.2. Antioxidant Activity

Antioxidant activity was evaluated by the radical scavenging capacity of the essential oils against the stable radical DPPH. The 50% inhibitory concentration (IC_50_) values were calculated and the IC_50_ values for both oils are presented in Table 2. Quercetin was employed as a positive control showing IC_50_ 10.47 ± 0.91 μg/mL.

### 3.3. Antimicrobial Activity

The antimicrobial activities of the essential oils from the leaves of both *A. schaueriana* specimens were evaluated against *C. albicans*, *A. brasiliensis*, *S. aureus*, *P. aeruginosa*, and *E. coli*. The growth inhibitions obtained for both oils, at the highest dose tested (217 µg/mL), are presented in Table 3. *P. aeruginosa* was the most sensitive organism for both oils, presenting the highest growth inhibition. 

## 4. Discussion

The two *A. schaueriana* specimens showed some variations in the essential oil compositions, as presented in Table 1. The Jureia-Itatins oil (**1**) was composed mostly of fatty acids (65.3%), having palmitic acid (46.5%) and myristic acid (11.6%) as major compounds, while in the Ilha do Cardoso oil (**2**), the main compounds were the phenylpropanoids eugenol (19.7%) and eugenol acetate (12.9%). In the second oil, fatty acids were only responsible for 18.1% of the oil composition, containing mainly palmitic acid (15.1%). The only *Avicennia* species that has had its essential oil contents previously analyzed was *A. marina*, and in this oil the main compounds were 2,6-bis(1,1-dimethylethyl)-4-methylphenol (BHT) (41.09%) and 2-phenyl-1,3-butadiene (24.56%) [12], while the fruit oils consisted mostly of methyl palmitate (41.9%) [13]. An interesting remark about these previous studies is the considerable amounts of synthetic compounds reported for both oils, such as BHT (41.9%), methyl *p*-vinylbenzoate (9.68%), and diethyl phthalate (1.2%) [12,13]. Some synthetic compounds were also found in both *A. schaueriana* oils, such as 1,2-dihydronaphthalene (2.4%–3.1%) and diisobutyl phthalate (0.7%), but not to the same extent as was reported for *A. marina*. The presence of these substances in the essential oils might be related to the constant exposure of mangrove plants to sea and river pollutants. It is not usual to detect these amounts of synthetic compounds in essential oils from inland plants, even when collected close to big cities as São Paulo. For example, the essential oil *from Chromolaena laevigata* (Lam.) R.M.King & H.Rob, collected at the São Paulo Botanical Gardens, consisted only of natural compounds [18]. 

The variability observed for the essential oil contents between the two *A. schaueriana* populations analyzed in this study might indicate the presence of distinct chemotypes for this species. Similar results were obtained with the extracts from *A. marina* leaves collected at two different Indian coasts, along the Arabic Sea and the Bay of Bengal, that were analyzed by GC-MS, showing a different chemical composition for these two populations [11,19]. The major constituents characterized in the Arabic Sea *A. marina* were the aromatic compounds 4-hydroxyphenylethanol and 3-methylbenzaldehyde (approximately 60%) [11], and the major compounds characterized in the Bay of Bengal *A. marina* were terpenes and fatty acids, such as phytol (13.3%), palmitic acid (10.70%), 6-hydroxy-3-oxo-α-ionol (8.6%) and palmitic acid ethyl ester (6.2%) [19]. Similar to the *A. schaueriana* oils, the *A. marina* extract differences could also be explained by the occurrence of chemotypes or by environmental factors that can also have an influence on chemical composition, such as the soil composition, temperature, relative humidity, sun exposure, wind regime and exposure to pathogens [20]. A more detailed study that involves other *A. schaueriana* populations is necessary in order to assess whether these differences are caused by environmental factors or by populational variations (chemotypes).

The *A. schaueriana* essential oils presented some radical scavenging activity with an IC_50_ of 0.90 mg/mL for oil **1** and 1.13 mg/mL for oil **2**, while quercetin, used as a positive control, showed an IC_50_ value of 10.47 ± 0.91 μg/mL. These results indicated that the essential oils were weaker antioxidants, when compared with quercetin. Oil **1** contained mainly fatty acids, palmitic and myristic acid, which are an important source of reserve energy and are components of the cell membrane in all living organisms. Additionally, in plants, fatty acid metabolic pathways also play significant roles in pathogen defense [21]. Saturated fatty acids can act as antioxidants or prooxidants [22]. The antioxidant activity of fatty extracts from 10 *Cephalaria* species, containing myristic and palmitic acids, showed IC_50_ values ranging from 3.77 to 15.12 mg/mL by the DPPH method [23]. The radical scavenging activity of *A. schaueriana* oils was higher than that found in *Cephalaria* species. Although oil **2** contained, as one of the major compounds, eugenol, a phenolic compound, the total oil activity was lower than that obtained for oil **1**. Pure eugenol showed a stronger antioxidant capacity than the positive control α-tocopherol (IC_50_ 33.85 μg/mL), and presented an IC_50_ of 16.06 μg/mL, using the same DPPH scavenging assay [24].

There is a large pool of publications describing the antioxidant activity of essential oils with active concentrations ranging from mg/mL to μg/mL. The antioxidant capability of 423 essential oils from 48 families by the DPPH assay was evaluated, and less than 5% of the tested oils showed antioxidant activity lower than 300 μg/mL [25]. The antioxidant capacity of essential oils is not very high compared with extracts and fractions rich in phenolic compounds. For example, the antioxidant activity of *Avicennia* species is reported for extracts and fractions, and the results were higher than those obtained for the *A. schaueriana* essential oils. The extracts from *A. marina* pneumatophores showed high antioxidant activity with an IC_50_ value of 21.22 μg/mL [26]. Furthermore, aqueous and ethanol extracts from *A. marina* barks, also rich in phenolic compounds, exhibited an important antioxidant activity with respective IC_50_ values of 112.7 μg/mL and 95.18 μg/mL [27]. 

The antimicrobial assay showed that both essential oils were not able to completely inhibit the growth in concentrations up to 217 μg/mL for all tested microorganisms. The highest activity was achieved against *P. aeruginosa* with 66.8 and 74.5 for oils **1** and **2**, respectively. This result can be interesting because, firstly, Gram-negative organisms possess an outer cell membrane impeding the diffusion of the hydrophobic essential oil constituents into the cell. Secondly, this is interesting to note because *P. aeruginosa* can also become resistant to certain antibiotics due to its ability to form biofilm, which consists of bacterial communities embedded in an exopolysaccharide matrix [28,29], making this organism a major cause of serious infection in hospitals. Higher concentrations could not be tested due the low amount of oil available. 

Normally, in screening studies with plant extracts and fractions, only MIC values lower than 100 μg/mL are considered active [28]. In general, most plant extracts present weak antimicrobial activity, with MIC values ranging in the mg/mL scale for crude extracts against various pathogens [28]. More specifically, in the case of essential oils, MIC values can range from 36,300 μg/mL to 0.001 μg/mL, and most of the activity is found in oxygenated terpenoids, but some hydrocarbons also might also exhibit some antimicrobial effects [28]. 

Essential oil **1**, containing mainly palmitic and myristic acids, and oil **2**, containing mostly eugenol, eugenol acetate and palmitic acid, showed an antimicrobial effect within the range already described for other oils. Furthermore, some fatty acids have already been studied for antimicrobial activity, but their MIC values were not as low as expected for high activity compounds [30,31]. The MIC values of myristic and palmitic acids were respectively 1600 μg/mL and >1600 μg/mL against *S. aureus* [30]. Palmitic acid was also evaluated against *C. albicans* with an MIC of 312.50 μg/mL [32], and with MIC values >256.42 μg/mL against *S. pyogenes*, *S. aureus*, *E. coli* and *P. aeruginosa* [33]. As can be seen in these references, the antimicrobial activity of myristic and palmitic acids always showed MIC values higher than 100 μg/mL. 

Eugenol has also been known for its antimicrobial properties. The antimicrobial activity of eugenol against *Propionibacterium acnes*, *Pityrosporum ovale*, *E. coli*, *C. albicans*, *S. aureus* MRSA, and *P. aeruginosa* showed MIC values of 50 μg/mL, 100 μg/mL, 800 μg/mL, 800 μg/mL, 1600 μg/mL, and 1600 μg/mL, respectively [34], and only for the two first microorganisms was this compound more active. The lower activity detected for the oil rich in eugenol (**2**) can be caused by interactions between the oil components leading to antagonist, additive or synergistic effects [35].

## 5. Conclusions

This was the first preliminary report of the constituents of *A. schaueriana* essential oils and their biological activities. The oils were composed mostly of fatty acids, but the Ilha do Cardoso population also presented eugenol and eugenol acetate as major compounds. As the two *A. schaueriana* populations analyzed presented differences in their essential oil contents, this might be an indication of different chemotypes for this species. More studies with other populations of *A. schaueriana* are necessary to prove if these differences are not only due to environmental factors. Concerning the biological activities of the essential oils, these oils presented no remarkable antioxidant or antimicrobial activities for their direct use as medicines. However, further studies are necessary to evaluate the antibacterial effect against *P. aeruginosa*, including synergism with other antimicrobials, due to its multidrug-resistant phenotypes and nosocomial status.

## Figures and Tables

**Table 1:** Composition of the essential oils from the leaves of *A. schaueriana* collected at Estação Ecológica Jureia-Itatins (**1**) and Parque Estadual da Ilha do Cardoso (**2**).

Compounds	RI ^a^	RI (lit.) ^b^	%	
			1	2
1-octen-3-one	977	977	-	0.7
1-octen-3-ol	981	979	1.8	4.3
*n*-nonanal	1103	1100	0.9	2.6
1,2-dihydronaphthalene	1151	1166	3.1	2.4
*n*-decanal	1205	1201	0.7	1.1
(2E)-decenal	1262	1263	2.5	3.4
N.I. 1: M^+^ 207, 119 (100%), 105 (55%), 91 (55%)	1326	-	0.7	0.8
N.I. 2: M^+^ 212, 43 (100%), 69 (91%), 109 (52%)	1332	-	-	1.1
eugenol	1350	1359	-	19.7
(E)-β-damascenone	1376	1384	3.6	3.5
N.I. 3: M^+^ 192, 159 (100%), 91 (73%), 105 (63%)	1382	-	3.7	4.8
N.I. 4: M^+^ 211, 157 (100%), 142 (70%), 172 (51%)	1385	-	-	1.1
N.I. 5: M^+^ 186, 157 (100%), 142 (72%), 172 (50%)	1388	-	-	1.3
N.I. 6: M^+^ 207, 44 (100%), 43 (82%), 159 (68%)	1399			0.8
methyl decyl ketone	1400	1389	1.0	-
N.I. 7: M^+^ 218, 159 (100%), 119 (99%), 91 (34%)	1406	-	4.8	6.8
*cis*-geranylacetone	1445	1436	-	1.3
N.I. 8: M^+^ 220, 135 (100%), 79 (67%), 150 (58%)	1456	-	1.0	1.2
N.I. 9: M^+^ 227, 133 (100%), 91 (80%), 105 (58%)	1459	-	-	0.7
eugenol acetate	1511	1522	-	12.9
caryophyllene oxide	1576	1583	-	1.6
epi-β-bisabolol	1667	1671	1.3	-
pentadecanal	1712	1713	-	1.0
myristic acid	1765	1770	11.6	3.0
hexahydrofarnesyl acetone	1839	1843	6.1	6.1
diisobutyl phthalate	1853	1866	0.7	0.7
pentadecanoic acid	1860	1870	1.0	-
N.I. 10: M^+^ 208, 84 (100%), 43 (72%), 85 (72%)	1882	-	0.7	-
musk ambrette	1895	1925	-	0.7
palmitic acid	1977	1960	46.5	15.1
linoleic acid	2128	2133	1.2	-
oleic acid	2136	2142	5.1	-
*n*-tetracosane	2497	2400	0.8	-
heptacosane	2697	2700	1.1	1.2
Total identified			89.1%	81.3%
Fatty acids			65.3%	18.1%
Hydrocarbons			1.9%	1.2%
Phenylpropanoids			-	32.6%
Oxygenated sesquiterpenes			1.3%	1.6%
Other			20.6%	27.7%
N.I.			10.9%	18.7%

^a^ Retention indices on DB-5 column; ^b^ Literature values [14,15]. N.I. = not identified.

**Table 2:** Antioxidant activity (IC_50_, mean ± CI_95_, *n* = 3) by DPPH method of the essential oil from the leaves of *A. schaueriana* collected at Estação Ecológica Jureia-Itatins (**1**) and Parque Estadual da Ilha do Cardoso (**2**).

Sample	IC_50_ (mg/mL)
**1**	0.90 ± 0.011 ^a^
**2**	1.13 ± 0.028 ^b^
	**IC_50_ (μg/mL)**
Quercetin	10.47 ± 0.91 ^c^

^a–c^ Mean values with different letters in the same column are significantly different (*p* < 0.05) according one-way Anova followed by multiple comparisons of Tukey’s test.

**Table 3:** Antimicrobial activity of the essential oil from the leaves of *A. schaueriana* collected at Estação Ecológica Jureia-Itatins (**1**) and Parque Estadual da Ilha do Cardoso (**2**).

Sample	Growth Inhibition (%) for 217 μg/mL (Mean ± CI_95_, *n* = 3)				
	*S. aureus* (ATCC 6538)	*E. coli* (ATCC 8739)	*P. aeruginosa* (ATCC 9027)	*C. albicans* (ATCC 10231)	*A. brasiliensis* (ATCC 16404)
**1**	-	13.5 ± 11.6	66.8 ± 4.8	15.3 ± 5.8	-
**2**	16.0 ± 10.6	33.7 ± 2.2	74.5 ± 9.3	12.1 ± 7.3	-
Ciprofloxacin (50 µg/mL) ^a^	95.0	100	100	N.A.	N.A.
Nystatin (50 µ g/mL) ^a^	N.A.	N.A.	N.A.	100	+

^a^ Concentration in each microplate well. N.A. = not applicable. − no inhibition; + total inhibition.

## Supplementary Materials

The following are available online at www.mdpi.com/2305-6320/4/2/26/s1, Figure S1: Mass spectrum of Non-identified compound **1** (N.I. 1) detected in the essential oil samples of *A. schaueriana* from Jureia; Figure S2: Mass spectrum of Non-identified compound **2** (N.I. 2) detected in the essential oil samples of *A. schaueriana* from Ilha do Cardoso; Figure S3: Mass spectrum of Non-identified compound **3** (N.I. 3) detected in the essential oil samples of *A. schaueriana* from Jureia; Figure S4: Mass spectrum of Non-identified compound **4** (N.I. 4) detected in the essential oil samples of *A. schaueriana* from Ilha do Cardoso; Figure S5: Mass spectrum of Non-identified compound **5** (N.I. 5) detected in the essential oil samples of *A. schaueriana* from Ilha do Cardoso; Figure S6: Mass spectrum of Non-identified compound **6** (N.I. 6) detected in the essential oil samples of *A. schaueriana* from Ilha do Cardoso; Figure S7: Mass spectrum of Non-identified compound **7** (N.I. 7) detected in the essential oil samples of *A. schaueriana* from Jureia; Figure S8: Mass spectrum of Non-identified compound **8** (N.I. 8) detected in the essential oil samples of *A. schaueriana* from Jureia; Figure S9: Mass spectrum of Non-identified compound **9** (N.I. 9) detected in the essential oil samples of *A. schaueriana* from Ilha do Cardoso; Figure S10: Mass spectrum of Non-identified compound **10** (N.I. 10) detected in the essential oil samples of *A. schaueriana* from Jureia..
