# Supplementary material for: Chemical Composition, Antimicrobial and Antioxidant Activities of Essential Oils from Two Avicennia schaueriana Stapf & Leechm. Ex Moldenke (Acanthaceae) Populations
Source: Medicines (Basel). 2017 May 1;4(2):26. doi: 10.3390/medicines4020026 (PMC5590062; doi:10.3390/medicines4020026)
Supplement: Supplementary file 1 [file medicines-04-00026-s001.pdf]

Supplementary Materials: Chemical Composition, Antimicrobial and Antioxidant Activities of Essential Oils from Two *Avicennia schaueriana* Stapf & Leechm. Ex Moldenke (Acanthaceae) Populations

Kamilla N. Machado, Telma M. Kaneko, Maria Cláudia M. Young, Cynthia Murakami, Inês Cordeiro and Paulo Roberto H. Moreno

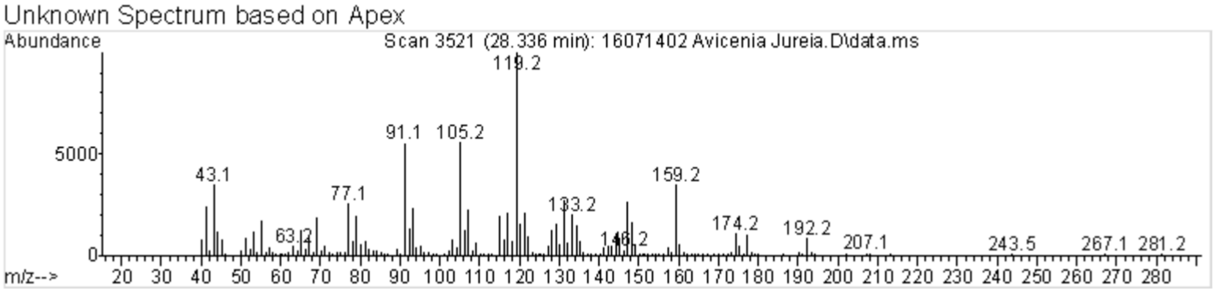

Figure S1. Mass spectrum of Non-identified compound 1 (N.I. 1) detected in the essential oil samples of *A. schaueriana* from Jureia.

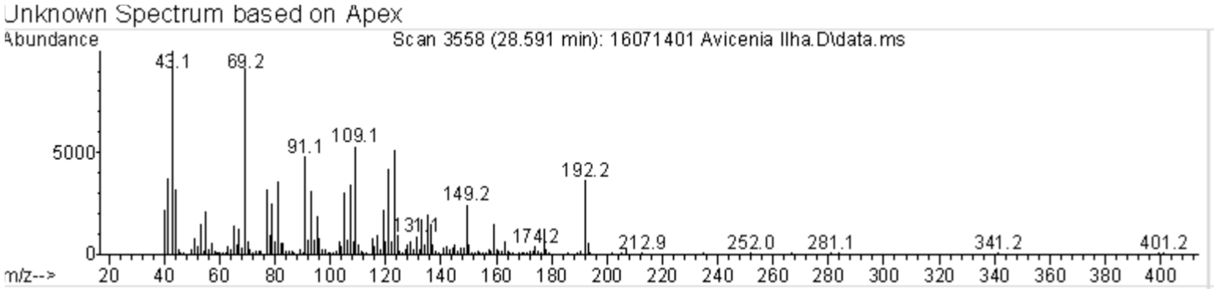

Figure S2. Mass spectrum of Non-identified compound 2 (N.I. 2) detected in the essential oil samples of *A. schaueriana* from Ilha do Cardoso.

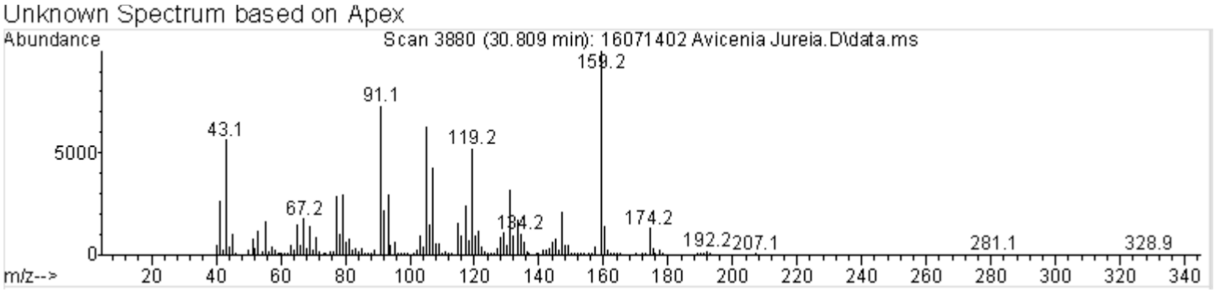

Figure S3. Mass spectrum of Non-identified compound 3 (N.I. 3) detected in the essential oil samples of *A. schaueriana* from Jureia.

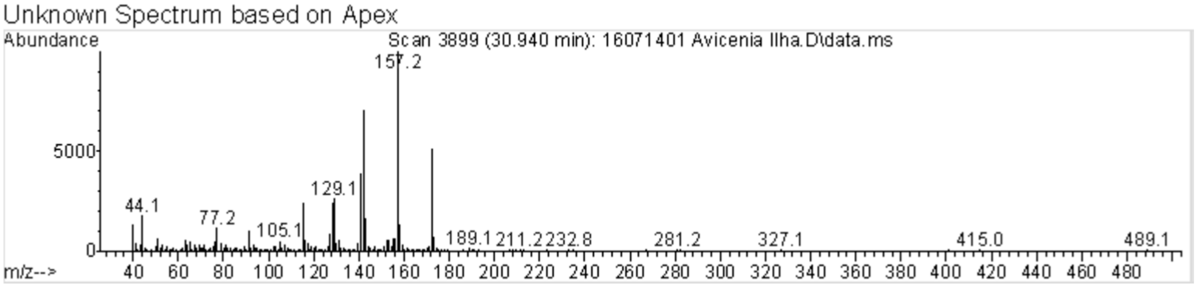

Figure S4. Mass spectrum of Non-identified compound 4 (N.I. 4) detected in the essential oil samples of *A. schaueriana* from Ilha do Cardoso.

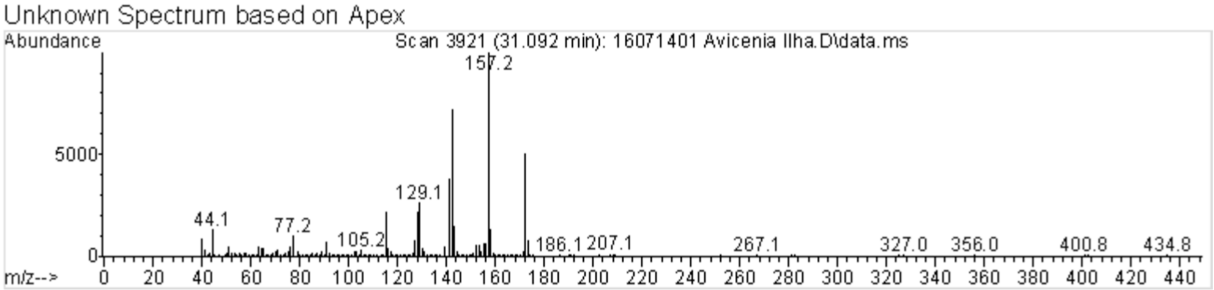

Figure S5. Mass spectrum of Non-identified compound 5 (N.I. 5) detected in the essential oil samples of *A. schaueriana* from Ilha do Cardoso.

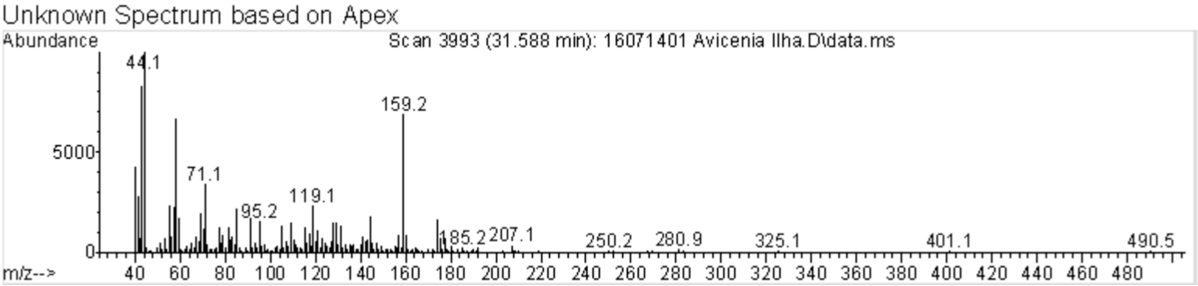

Figure S6. Mass spectrum of Non-identified compound 6 (N.I. 6) detected in the essential oil samples of *A. schaueriana* from Ilha do Cardoso.

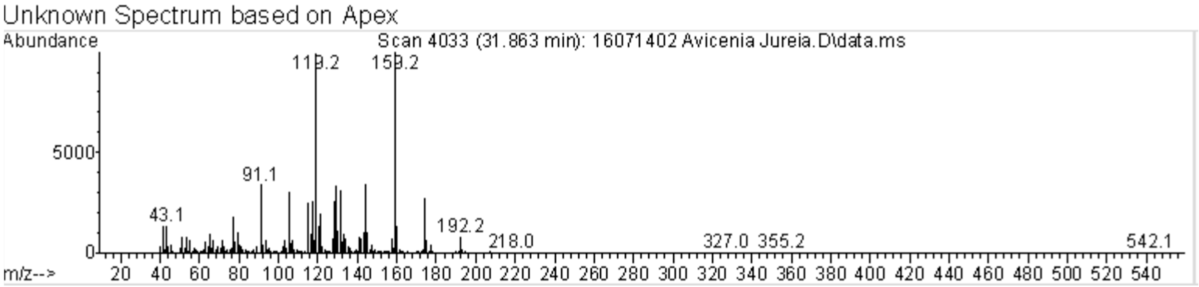

Figure S7. Mass spectrum of Non-identified compound 7 (N.I. 7) detected in the essential oil samples of *A. schaueriana* from Jureia.

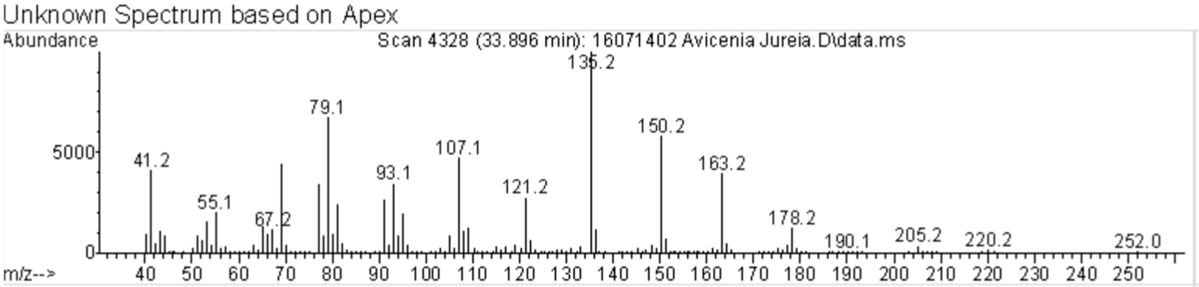

Figure S8. Mass spectrum of Non-identified compound 8 (N.I. 8) detected in the essential oil samples of *A. schaueriana* from Jureia.

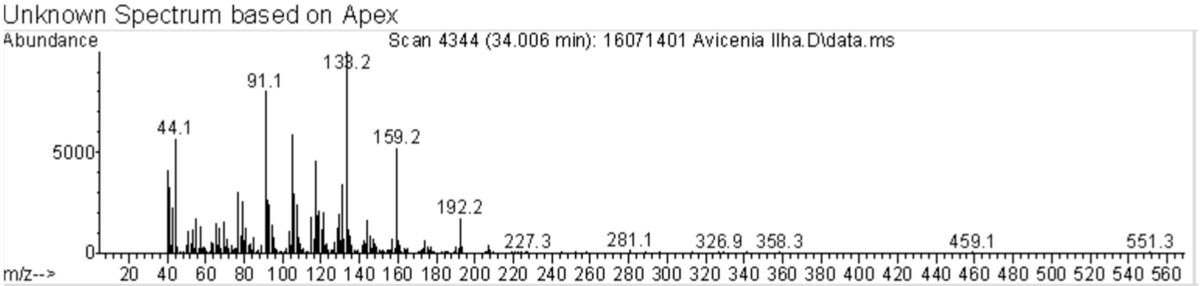

Figure S9. Mass spectrum of Non-identified compound 9 (N.I. 9) detected in the essential oil samples of *A. schaueriana* from Ilha do Cardoso.

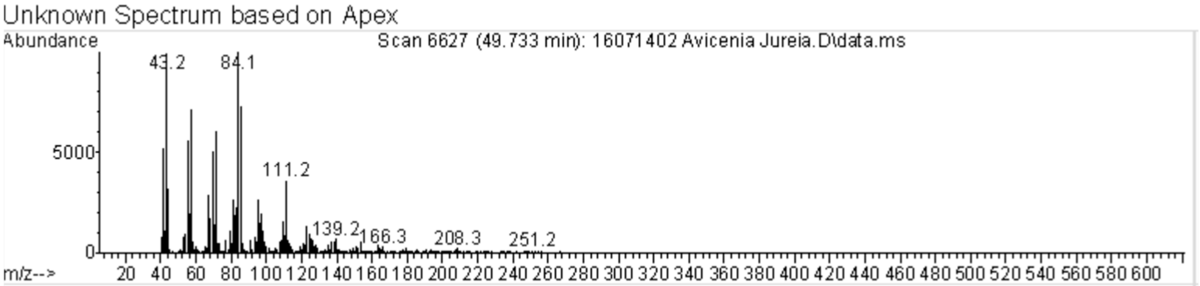

Figure S10. Mass spectrum of Non-identified compound 10 (N.I. 10) detected in the essential oil samples of *A. schaueriana* from Jureia.
